# B cell antigen receptor-induced activation of an IRAK4-dependent signaling pathway revealed by a MALT1-IRAK4 double knockout mouse model

**DOI:** 10.1186/1478-811X-9-6

**Published:** 2011-03-11

**Authors:** Almut Dufner, Wolfgang W Schamel

**Affiliations:** 1 Campbell Family Institute for Breast Cancer Research, 620 University Avenue, Toronto, Ontario, M5G 2C1, Canada; 2 Max-Planck-Institute of Immunobiology and Epigenetics, Stübeweg 51, 79108 Freiburg, Germany; 3 Department of Molecular Immunology, Institute for Biology III, Albert-Ludwigs University Freiburg, Stübeweg 51, 79108 Freiburg, Germany; 4 Centre of Chronic Immunodeficiency, University Medical Centre Freiburg, Breisacher Straße 177, 79106 Freiburg, Germany; 5 Centre for Biological Signaling Studies (BIOSS), Albert-Ludwigs University Freiburg, 79104 Freiburg, Germany; 6 University Hospital Freiburg, Department of Neuropathology, Breisacher Straße 64, 79106 Freiburg, Germany

## Abstract

**Background:**

The B cell antigen receptor (BCR) and pathogen recognition receptors, such as Toll-like receptor 4 (TLR4), act in concert to control adaptive B cell responses. However, little is known about the signaling pathways that integrate BCR activation with intrinsic TLR4 stimulation. Antigen receptors initialize activation of the inducible transcription factor nuclear factor-κB (NF-κB) via recruitment of the membrane-associated guanylate kinase caspase recruitment domain protein 11 (CARD11), the adapter molecule B cell CLL/lymphoma 10 (BCL10), and the "paracaspase" mucosa-associated lymphoid tissue lymphoma translocation gene 1 (MALT1) into lipid rafts. Upon BCR triggering, this activation strictly depends on BCL10, but not on MALT1, leading to the hypothesis that a MALT1-independent NF-κB activation pathway contributes to BCR-induced NF-κB activation downstream of BCL10. The identity of this pathway has remained elusive.

**Results:**

Using genetic and biochemical approaches, we demonstrate that the IRAK4- and IRAK1-dependent TLR signaling branch is activated upon BCR triggering to induce partial NF-κB activation. BCR-induced MALT1-independent IκB degradation and B cell proliferation were inhibited in MALT1/IRAK4 double knockout B cells. Moreover, IRAK1 was recruited into lipid rafts upon BCR stimulation and activated following transient recruitment of IRAK4.

**Conclusion:**

We propose that the observed crosstalk between BCR and TLR signaling components may contribute to the discrimination of signals that emanate from single and dual receptor engagement to control adaptive B cell responses.

## Background

Activation and survival of B cells in response to antigen receptor (AgR) engagement depends on the activation of the inducible transcription factor NF-κB. BCR-induced NF-κB activation is mediated by components of the so-called CBM signaling complex. The CBM complex consists of the CARD-containing membrane-associated guanylate kinase CARD11, the CARD-containing adaptor protein BCL10, and the death domain (DD)-containing "paracaspase" MALT1 [1-5]. Complex assembly and the recruitment of downstream effectors are triggered by a receptor-proximal tyrosine phosphorylation cascade that leads to the activation of protein kinase C-β (PKC-β) [6,7]. PKC-β phosphorylates a linker region in the adaptor molecule CARD11, which enables CARD11 to recruit BCL10 and MALT1 into lipid rafts [8]. BCL10 and MALT1 then mediate activation of the IKK complex that induces degradation of IκB proteins, the inhibitors of NF-κB that retain it in the cytoplasm, which ultimately leads to the activation of NF-κB [9]. This process requires lysine 63-linked polyubiquitination events that involve the E3-ligase tumor necrosis factor receptor-associated factor 6 (TRAF6) and mediate complex formation between components of the CBM complex, TRAF6, transforming growth factor β-activated kinase 1 (TAK1) and the IKK complex [10-13].

Paradoxical to the established requirement of MALT1 for T cell AgR (TCR)-mediated proliferation and NF-κB activation, BCR-driven proliferation and IκB degradation are reduced, but not abrogated in MALT1-deficient B cells, even though the impact on B cell proliferation was contradictory among previous reports [3-5,14]. In contrast, BCL10-deficient B cells exhibit complete inhibition of proliferation and IκB degradation in response to BCR engagement [3,4,14]. These findings have been attributed to the differential activation of the NF-κB subunits RelA and c-Rel. In BCL10-/- B cells both subunits remain bound to undegraded IκBα following BCR activation, whereas in MALT1-/- cells only the activation of c-Rel-containing NF-κB dimers is affected [3]. These results suggested the presence of an alternative, MALT1-independent BCR-induced NF-κB activation pathway capable of activating RelA downstream of BCL10.

TLRs are responsible for the recognition of pathogen-associated molecular patterns expressed by extracellular pathogens. Toll-like receptor 4 (TLR4) is the prototypic TLR that recognizes lipopolysaccharide (LPS) derived from the outer membrane of gram-negative bacteria [15]. It relays signals to NF-κB via two pathways, one branch involving the Toll-interleukin-1 receptor (TIR) domain-containing adapter proteins TIRAP and MyD88, which in turn recruit the DD-containing kinases interleukin receptor-associated kinase 4 (IRAK4) and IRAK1. IRAK1 then activates NF-κB in a signaling pathway that utilizes many components of AgR-induced NF-κB activation downstream of MALT1. Alternatively, TLR4 activates NF-κB via the TIR domain-containing adaptor inducing interferon-β (TRIF) and receptor-interacting protein 1 (RIP1) [16].

One study using MALT1-/- mice suggested that MALT1 is required for TLR4-induced B cell proliferation [5]. A parallel study did not confirm a defect in TLR4 signaling in MALT1-/- B cells [4]. This discrepancy could be due to different MALT1 knockout (KO) strategies, which may point to a crosstalk between BCR- and TLR4-mediated NF-κB activation in B cells. Indeed, previous reports have indicated that the BCL10-MALT1 pathway interacts with TLR4 signaling. BCL10 has been shown to be important for LPS signaling to NF-κB in marginal zone B cells [17]. In addition, it has been reported that BCL10 and MALT1 are part of NF-κB-inducing signaling complexes downstream of TLR4 receptors in macrophages [18,19]. Conversely, IRAK4 has been suggested to play a critical role in TCR-induced NF-κB activation upstream of PKCθ [20]. This hypothesis has recently been challenged by others, however, who could not confirm a defect in TCR-induced *ex vivo *proliferation of IRAK4-/- T cells [21,22].

Signaling by pathogen recognition receptors (PRRs) of the innate immune system has been shown to be essential for the initiation of efficient adaptive T and B cell responses [23,24]. We have demonstrated that induced proximity of the BCR and the PRR TLR4 by antigen-coupled LPS results in a synergistic increase in B cell activation [25]. The finding that TLR4 may associate with the BCR via its transmembrane domain [26] adds further evidence to a receptor-proximal signaling crosstalk.

Here we examined whether an IRAK4-dependent mechanism is required for MALT1-independent BCR signaling downstream of BCL10. To test this hypothesis, we generated MALT1/IRAK4 double knockout (DKO) bone marrow (BM) chimera and compared their BCR activation potential to that of MALT1 single KO BM chimera. In addition, we monitored the lipid raft recruitment and activation of IRAK4 and IRAK1 in response to BCR activation. Our results demonstrate that mediators of the TLR4 signaling pathway to NF-κB are engaged upon BCR activation to contribute to B cell proliferation.

## Results

### MALT1 and IRAK4 independently mediate antigen receptor-induced B cell proliferation

**Figure 1:**
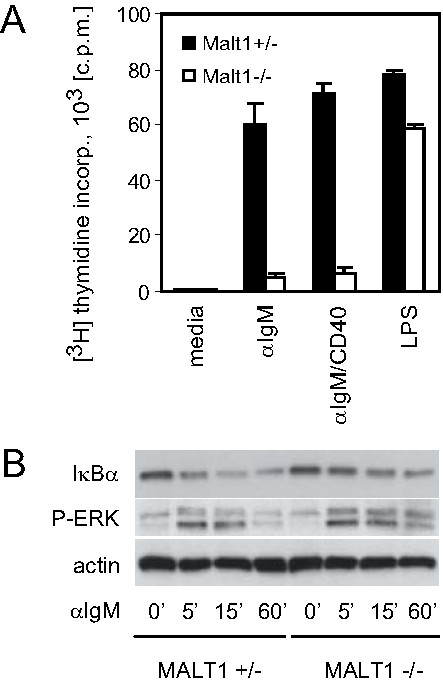
**Activation of BCR signaling in MALT1-/- B cells**. (*A*) B cell proliferation. Splenic MALT1+/- and MALT1-/- B cells were stimulated for 36 h with soluble anti-IgM, anti-IgM plus anti-CD40, or LPS. Proliferation was measured by [^3^H]thymidine incorporation. Results are presented as the mean [^3^H]thymidine incorporation ± S.D. for triplicate samples after an 8 h pulse and are 1 trial representative of at least 3 independent experiments. (*B*) IκBα degradation. Splenic MALT1+/- and MALT1-/- B cells were stimulated for the indicated times with anti-IgM. IκBα degradation, ERK1/2 phosphorylation (P-ERK), and actin levels were determined by Western blotting.

To reassess the role of MALT1 in B cell activation, we purified splenic B cells from MALT1+/- and MALT1-/- mice and stimulated them with anti-IgM antibodies, or anti-IgM plus anti-CD40, or LPS. In agreement with earlier studies, IgM-BCR-induced NF-κB activation translated into partial long term B cell proliferation in MALT1-/- B cells [3,4], although the observed inhibition of proliferation was more severe than suggested by some of the earlier reports [4,14] (Figure 1A). Moreover, LPS-induced MALT1-/- B cell proliferation was not significantly affected (Figure 1A and data not shown). As shown previously [4,5], MALT1-/- B cells exhibited substantial, yet partially impaired short term degradation of IκBα in response to BCR engagement (Figure 1B). Anti-IgM-induced activation of the MAP kinases ERK1 and ERK2 was not affected in the absence of MALT1 (Figure 1B). These results confirm that BCR-stimulated IκBα degradation is at least in part MALT1-independent.

**Figure 2:**
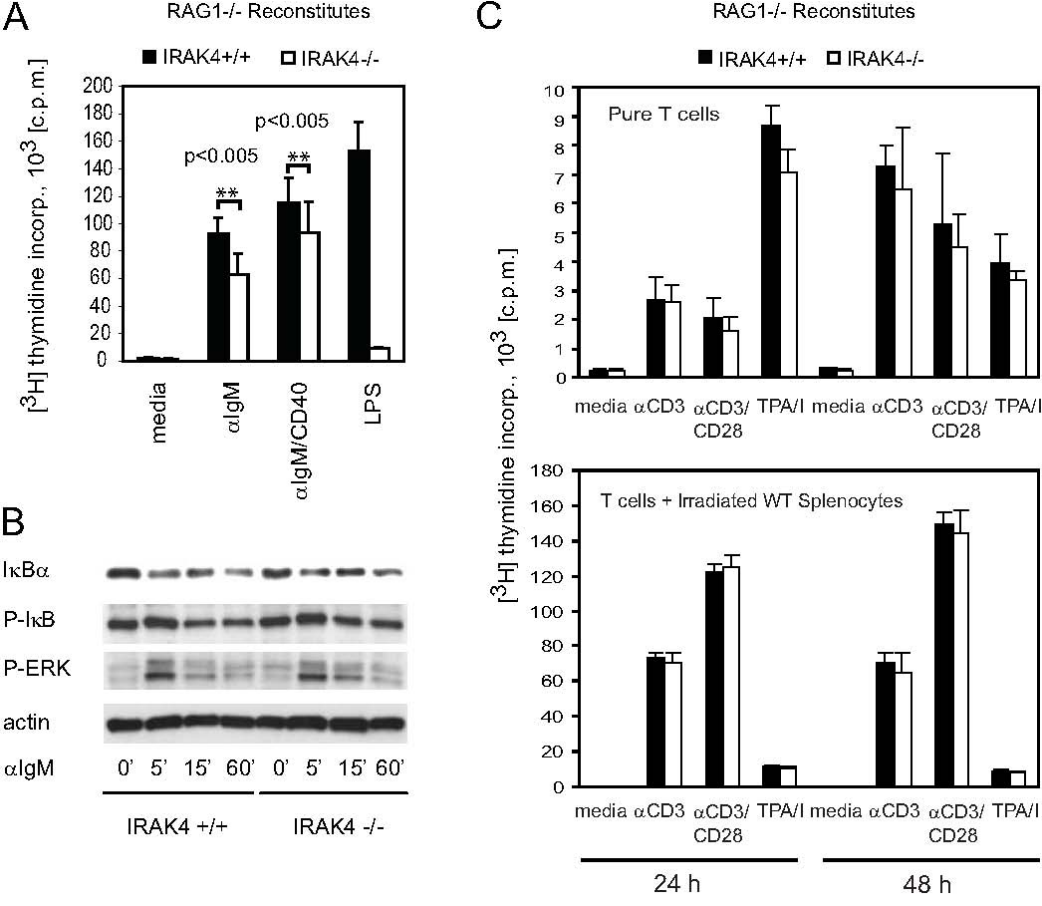
**B and T cell activation in the absence of IRAK4**. (*A*) Partial defect in BCR-induced B cell expansion in the absence of IRAK4. Splenic IRAK4+/+ and IRAK4-/- B cells from RAG1-/- mice reconstituted with IRAK4+/+ or IRAK4-/- BM cells were stimulated as in Figure 1A. Proliferation was measured by [^3^H]thymidine incorporation. Results are presented as the mean [^3^H]thymidine incorporation ± S.D. for 6 independent experiments after an 8 h pulse. The data were analyzed using the dependent (paired samples) t-test. (*B*) IκBα degradation after anti-IgM stimulation of IRAK4-/- B cells. Splenic IRAK4+/+ and IRAK4-/- B cells from reconstituted RAG1-/- mice were stimulated as in Figure 1B and IκBα degradation and phosphorylation (P-IκBα), ERK1/2 phosphorylation and actin levels were analyzed by Western blotting. (*C*) Normal TCR-induced proliferation of IRAK4-/- T cells. Upper panel: Purified IRAK4+/+ and IRAK4-/- T cells from reconstituted RAG1-/- mice were stimulated for the indicated times with anti-CD3 in the absence or presence of anti-CD28, or with TPA/Ca^2+ ^ionophore A23187 (I). Lower panel: Purified T cells were mixed with irradiated WT splenocytes and stimulated as in the upper panel. For both sets of panels, proliferation was measured as in (*A*). Results are presented as the mean [^3^H]thymidine incorporation ± S.D. for 3 independent experiments after an 8 h pulse.

Next, we examined whether B cell proliferation in response to IgM-BCR stimulation or through both IgM-BCR and CD40 is affected in IRAK4-/- mice. To generate mice with lymphocyte-specific deletion of IRAK4, we reconstituted RAG1-/- mice with BM from IRAK4-/- mice or from WT mice. Consistent with a role for IRAK4 in BCR signaling, IRAK4-/- B cells exhibited partial hypoproliferation in response to anti-IgM stimulation in the absence or the presence of anti-CD40 (Figure 2A). Thymidine incorporation into IRAK4-/- B cells, which was monitored in six independent experiments, was reduced by approximately 30% compared to WT B cells (Figure 2A). LPS-induced B cell proliferation was almost completely inhibited in the absence of IRAK4. In line with the minor defect in anti-IgM-induced B cell expansion, the corresponding short term IκBα degradation and phosphorylation appeared to be normal in IRAK4-/- B cells (Figure 2B). Again, ERK activation was unaffected. In summary, these results indicate that IRAK4 is important for efficient BCR-induced B cell expansion.

In parallel experiments, we monitored the activation potential of WT and IRAK4-deficient T cells from lymph nodes of the same reconstituted RAG1-/- mice (Figure 2C). It has been argued that IRAK4 is specifically required for T cell activation by weak signals such as stimulation by soluble anti-CD3 antibody [27]. To distinguish between quantitative and qualitative differences in stimulation signals, we exposed T cells to either soluble antibodies and agonists alone or in the presence of irradiated WT splenocytes to provide additional co-stimulatory signals. Independently of signal strength, we did not observe any significant differences in T cell activation by stimulation with anti-CD3 in the absence or presence of anti-CD28, or with TPA plus Ca^2+ ^ionophore A23187 (Figure 2C). In conclusion, IRAK4 specifically contributes to the AgR-mediated activation of B cells.

**Figure 3:**
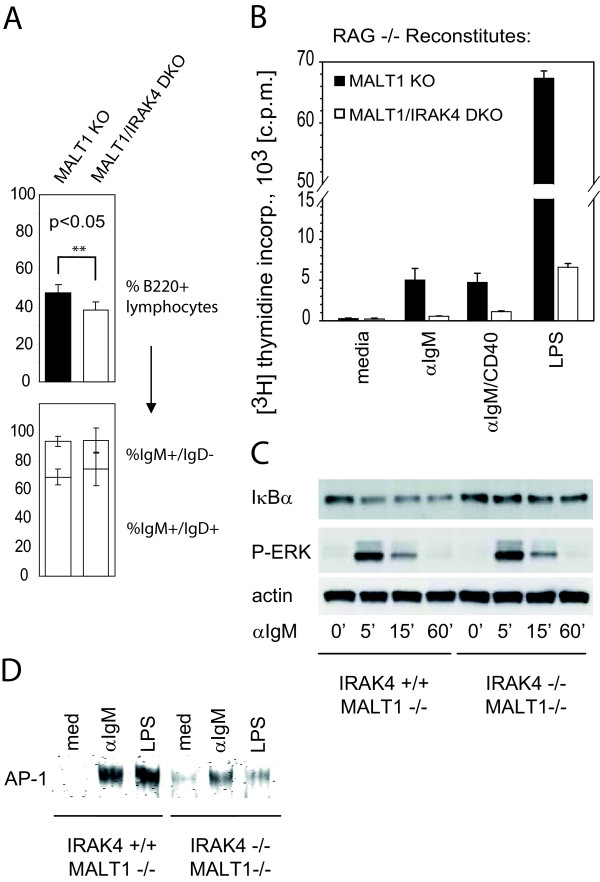
**B cell activation defects in the absence of MALT1 and IRAK4**. (*A*) Flow cytometric analysis of splenic MALT1 KO and MALT1/IRAK4 DKO lymphocytes. Upper panel: Percentages of B220^+ ^lymphocytes; the data were analyzed using the dependent (paired samples) t-test (n = 5). Lower Panel: Percentages of IgM single or IgM/IgD double positive B220^+ ^splenocytes. (*B*) Splenic MALT1 KO and IRAK4/MALT1 DKO B cells from RAG1-/- mice reconstituted with MALT1 KO or MALT1/IRAK4 DKO BM cells were stimulated as in Figure 1A and the proliferative response was measured accordingly. Results are presented as the mean [^3^H]thymidine incorporation ± S.D. for triplicate samples after an 8 h pulse and are 1 trial representative of at least 3 independent experiments. (*C*) Splenic MALT1 KO and MALT1/IRAK4 DKO B cells from reconstituted RAG1-/- mice were stimulated and analyzed by Western Blotting as in Figure 1B. (*D*) Splenic MALT1 KO and MALT1/IRAK4 DKO B cells from reconstituted RAG1-/- mice were stimulated with soluble anti-IgM or LPS for 6 hours and AP-1 activation was determined by Electrophoretic Mobility Shift Assay (EMSA).

To test whether IRAK4 is involved in BCR signaling in a manner that is independent of MALT1, we compared the BCR activation potential of MALT1-/- B cells to that of B cells lacking both MALT1 and IRAK4. MALT1/IRAK4 DKO mice did not display any gross developmental defects, but died within one week after weaning (not shown). Presumably, young MALT1/IRAK4 DKO mice succumb to infections due to the lack of breast feeding-mediated passive immunity after weaning. The percentage of splenic MALT1/IRAK4 DKO B lymphocytes (B220^+^) was slightly reduced compared to the percentage of MALT1 KO B220^+ ^lymphocytes (Figure 3A). The mean difference between single and double KO littermates amounts to 9.2% (std. dev.: 5.7%; P < 0.05). In contrast, percentages of splenic IgM^+^IgD^- ^and IgM^+^IgD^+ ^B cells were not significantly altered (Figure 3A). Likewise, there were no significant differences in the percentages of follicular or marginal zone B cells, or pre/pro B cells in the BM between the two genotypes (data not shown).

For the B cell activation experiments, we reconstituted RAG1-/- mice with BM from MALT1 KO or MALT1/IRAK4 DKO mice. Isolation and stimulation of splenic B cells with anti-IgM or anti-IgM plus anti-CD40 revealed severe inhibition of the expansion of MALT1-deficient B cells if IRAK4 was also absent (Figure 3B). In line with this result, degradation of IκB was diminished in MALT1/IRAK4 DKO cells compared to MALT1 KO cells (Figure 3C). Activation of ERK was not affected by the absence of IRAK4.

We have also monitored JNK activation for further analysis of the observed BCR-TLR4 signaling crosstalk (Figure 3D). Similar to NF-κB activation, JNK activation has been reported to be completely impaired in MALT1-/- T cells that have been stimulated with PMA/ionomycin in one report [4]. These data are in conflict with another report, however, showing normal JNK phosphorylation in PMA/ionomycin-stimulated MALT1-/- T cells [5]. In MALT1-/- B cells, BCR-mediated activation of the JNK downstream effector AP-1 is not affected [4]. We have monitored AP-1 activation in MALT1 single KO and MALT1/IRAK4 double KO B cells that have been stimulated with anti-IgM for 6 hours. Consistent with a role for IRAK4 in MALT1-independent AP-1 activation, our data demonstrate that AP-1 activation is partially inhibited in anti-IgM stimulated MALT1/IRAK4 double KO B cells compared to MALT1 single KO B cells. As expected, AP-1 activation by LPS was blocked in the absence of IRAK4 [28].

These data establish IRAK4 as a modulator of BCR signaling that is independent of MALT1 function.

### IRAK4 and IRAK1 are activated by BCR engagement

**Figure 4:**
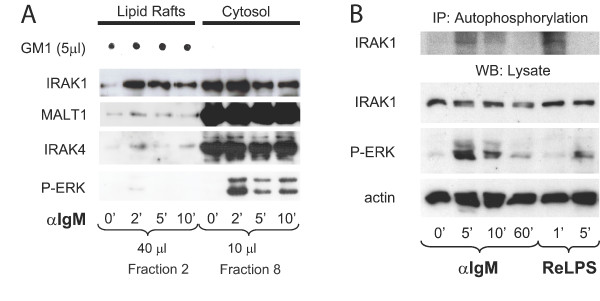
**Lipid raft recruitment and activation of IRAK1 and IRAK4**. (*A*) Lipid raft recruitment of IRAK1 and IRAK4. Splenic WT B cells were left unstimulated or were stimulated for the indicated times with anti-IgM. Lysates were separated by sucrose-gradient ultracentrifugation and the indicated amounts of the collected fractions were analyzed by Western blotting with antibodies to IRAK1, MALT1, IRAK4, and phospho-ERK. To identify the lipid raft containing samples, 5 μl of each fraction were spotted onto a nitrocellulose membrane and GM1 was visualized with FITC-labeled Cholera toxin. *(B) *Activation of IRAK1 upon BCR triggering. Splenic WT B cells were stimulated for the indicated times with anti-IgM or ReLPS. The kinase activity of immunoprecipitated IRAK1 was measured by an autophosphorylation assay. IRAK1, phospho-ERK1/2, and actin were quantified by Western blotting of the lysates. IP, immunoprecipitation; WB, Western blotting.

To examine whether the IRAK4-dependent pathway is directly involved in BCR signaling to NF-κB, we asked whether components of this signaling cascade are recruited into lipid rafts after BCR engagement. To this end, we isolated lipid rafts by sucrose gradient ultracentrifugation and controlled the experiment by Western blotting against phospho-ERK and MALT1 and dot blotting against the raft-resident ganglioside GM1. We observed substantial accumulation of IRAK1 in lipid rafts of splenic WT B cells that were stimulated with anti-IgM for at least 2 minutes (Figure 4A). In contrast, our time course experiment revealed that IRAK4 and MALT1 get recruited into lipid rafts transiently. At two minutes post BCR stimulation, IRAK4 raft-accumulation was detected that declined after 5 minutes. In contrast, membrane-associated IRAK1 levels remained elevated for at least 10 minutes post BCR stimulation. In the context of TLR signaling IRAK1 gets recruited, phosphorylated and activated by IRAK4 to relay signals to NF-κB. Hence, our results imply that BCR activation not only causes recruitment of IRAK4 and IRAK1 into lipid rafts, but also leads to IRAK1 activation.

To test whether the activation state of IRAK1 changes upon BCR triggering, we monitored its autophosphorylation in IgM-BCR-stimulated B cells. It should be noted, however, that the functional relevance of the kinase activity of IRAK1 is still uncertain [29]. In agreement with a direct effect of BCR stimulation on IRAK1 activity, we observed a transient increase of IRAK1 autophosphorylation in response to anti-IgM stimulation (Figure 4B). As a control, we treated B cells with the "rough(R)-form" ReLPS, which is a more potent activator of TLR4 in B cells than the conventional "smooth form" S-LPS [25,30]. ReLPS stimulation resulted in a more rapid autophosphorylation of IRAK1 than anti-IgM stimulation. Unlike ERK activation, which peaked after 5 minutes of ReLPS stimulation, IRAK1 autophosphorylation was stimulated within the first minute of exposure and was back to basal levels five minutes post stimulation.

In summary, these results demonstrate that the BCR relays signals to IRAK4 and IRAK1, which ultimately leads to the lipid raft recruitment and enzymatic activation of IRAK1.

## Discussion

**Figure 5:**
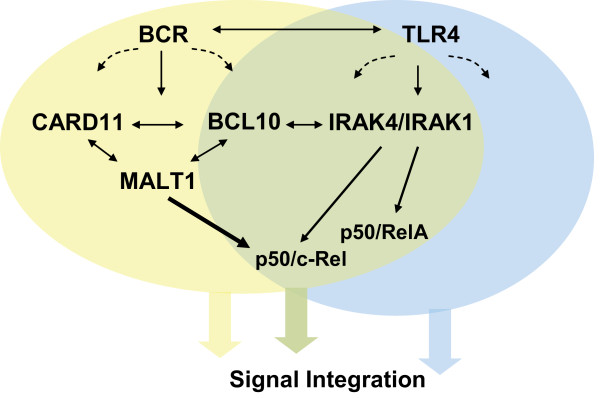
**NF-κB activation in B cells**. Proposed signal integration during BCR and TLR4 crosstalk. Differential (sequential) complex configuration is indicated by the use of complementary colors.

Using genetic and biochemical approaches, we show that IRAK4 and IRAK1 are involved in BCR-induced NF-κB activation (Figure 5). Until now, these kinases were known for being definitive mediators of signaling by members of the IL-1 receptor and TLR families [31]. More recently, they have been suggested to transmit signals to NF-κB in response to activation of the tumor necrosis factor receptor (TNFR) familiy member transmembrane activator and calcium modulator and cyclophilin ligand interactor (TACI) to control T cell-independent class-switch recombination [32]. It is conceivable that the observed involvement in BCR signaling is mediated by direct interactions with the CBM complex or by an adaptor molecule that bridges BCL10 recruitment to the recruitment of IRAK4-containing complexes into lipid rafts. Pellino proteins have been shown to function as E3 ubiquitin ligases that target IRAK1 to regulate the recruitment of the IKK complex [33]. Interestingly, one group found that both IRAK1 and Pellino-2 directly interact with BCL10 [18,19]. They suggest that IRAK1 cooperates with BCL10 and MALT1 in the TLR4 signaling pathway to NF-κB in RAW264.7 macrophages [19]. Pellino-2 transcripts are not detected in adult mouse spleen, however [34]. Further investigation into the identification and analysis of CBM-interacting proteins is required to establish the precise mechanism that couples pathways triggered by the BCR to components of TLR-induced signal transduction cascades.

Activation of the IRAK4/IRAK1 axis by the BCR is not sufficient to overcome the defects in signaling and cellular expansion caused by the absence of a functional MALT1 subcomplex, however. Deletion of MALT1 may lead to the generation of a non-functional complex, in which c-Rel-containing dimers are kept in an inactive state. Likewise, IRAK4/IRAK1 signals may specifically allow nuclear translocation and activation of the RelA-containing subset of NF-κB transcription factors independently of MALT1's organizer function that has been hypothesized to specifically promote the interaction of the IKK complex with c-Rel-bound IκB [3]. Third, BCR-activated IRAK4 may induce both c-Rel and RelA-containing NF-κB dimers with slower kinetics than MALT1 thereby compromising more efficient B cell activation in the absence of a TLR4 signal.

Vice versa, there appears to be residual IκB degradation even in the absence of MALT1 and IRAK4. Similar to MALT1, caspase 8 is critical for efficient TCR-mediated cell expansion, IL-2 production [35], and short term NF-κB activation [36], but not for anti-IgM-induced proliferation of B cells [37]. It has been suggested that MALT1-directed cleavage of the caspase 8 regulator c-Flip by caspase 8 contributes to NF-κB activation after TCR-induced recruitment of caspase 8 into lipid rafts [38]. The latter model places caspase 8 downstream of the MALT1 paracaspase domain in TCR signaling, however, which contradicts the model that puts the NF-κB signaling bifurcation point at the level of BCL10. It remains to be clarified, whether caspase 8 plays a role in the TRIF-dependent branch of TLR4-induced NF-κB activation. This pathway is mediated by the IRAK family-related kinase RIP1, another candidate that may participate in a crosstalk between receptors of innate and adaptive immunity. Such crosstalk has already been proposed for the RIP kinase family member RIP2, a mediator of NF-κB activation by the intracellular PRRs for bacterial peptidoglycan nucleotide-binding oligomerization domain containing 1 (NOD1) and NOD2. A previous report indicated that RIP2 directly interacts with BCL10, and T cell activation was initially reported to be impaired in RIP2-/- cells [39]. Even though these findings have later been attributed to undetected NOD signaling that is important for priming of an adaptive immune response [24], they may point to a common theme of B cell-intrinsic crosstalk between receptor proximal BCR and PRR signaling.

More recently, MALT1 has been shown to be required for activation of the alternative NF-κB signaling pathway by B cell activation factor of the TNF family (BAFF), which is required for BAFF receptor-mediated B cell survival [14]. Coupled BCR-TLR4 recognition on the other hand may promote synergistic effects on B cell activation [25] via recruitment of additional signaling molecules into a dual-receptor proximal supercomplex (Figure 5).

## Conclusions

Signaling modules that activate NF-κB downstream of TLR4 are engaged upon BCR activation. Our findings have important implications for the integration of signals downstream of the BCR and the subset of TLRs that target IRAK4 to mediate adequate antibody responses.

## Methods

### Mouse strains

The MALT1-/- and IRAK4-/- mice were in the C57BL/6 background and have been described previously [4,28]. All mice were maintained at animal facilities of the Ontario Cancer Institute or the Max-Planck-Institute of Immunobiology and Epigenetics according to institutional protocols.

### RAG1-/- reconstitution

RAG1-/- mice were γ-irradiated with a dose of 600 rad and penicillin/streptomycin was added to the drinking water. The next day, the mice were injected with 2.5 - 5 × 10^6 ^BM cells. BM from tibias and femurs was obtained by flushing with PBS containing 1% FCS and depleted of red blood cells (RBCs) using RBC lysis buffer (Sigma Aldrich). The reconstituted mice were kept on antibiotics for 2 weeks and analyzed after 2-3 months.

#### T and B cell isolation, stimulation and proliferation assays

T and B cells were maintained in RPMI 1640 medium containing 10% FCS, 100 μg ml^-1 ^penicillin/streptomycin, and 50 μM β-mercaptoethanol. T cells were purified using IMag Streptavidin Particles Plus - DM (BD Pharmingen). For the proliferation and stimulation experiments, B cells were isolated with biotinylated anti-CD19 antibody (BD Pharmingen) and IMag Streptavidin Particles Plus - DM as described before [40]. For T cell proliferation assays, 10^5 ^T cells were stimulated in 96-well U-bottom plates in the presence or absence of 10^5 ^irradiated (30 Gy) unfractionated WT splenocytes. For T cell stimulation, anti-CD3 (145-2C11, BD Pharmingen) and anti-CD28 (553294, BD Pharmingen) were used at a concentration of 1 μg ml^-1^. Alternatively, T cells were stimulated with 10 ng ml^-1 ^TPA (Sigma) plus 50 ng ml^-1 ^Ca^2+ ^ionophore A23187 (Sigma). For B cell proliferation assays, purified B cells (5-10 × 10^4^) were stimulated by incubation with 5 μg ml^-1 ^anti-IgM [F(ab')_2 _fragment, 115-006-020, Jackson ImmunoResearch Laboratories] with or without 5 μg ml^-1 ^anti-CD40 (553787, BD Pharmingen), or 20 μg ml^-1 ^ultra pure *E. coli *LPS (InvivoGen). [^3^H]thymidine incorporation was measured as described before [40]. For short term stimulation experiments, B cells were activated by preincubation with anti-IgM (10 μg ml^-1^) for 30 min on ice and stimulated for the times indicated at 37°C. LPS short term stimulation was performed with 25 μg ml^-1 ^ReLPS [30].

#### Immunoprecipitation, kinase assay, Western blotting and EMSA

Cells were lysed as described previously [41]. Protein lysates were precleared with protein G sepharose 4B (Sigma) and subsequently proteins were immunoprecipitated by combining soluble cell lysate with protein G sepharose 4B coupled to the mouse monoclonal IRAK1 (F-4) antibody (Santa Cruz Biotechnology). After 2 h incubation at 4°C with constant rotation, immunoprecipitates were washed 3 times with lysis buffer and once with kinase buffer (25 mM Tris-HCl, pH 7.5; 10 mM MgCl_2_; 5 mM β-glycerophosphate; 1 mM sodium vanadate). Kinase assays were performed in kinase buffer in the presence of 10 μCi [γ^32^P]ATP and 10 μM cold ATP. Reactions were incubated at 37°C for 30 min and stopped by addition of SDS-PAGE loading buffer. Western blot analysis was performed using antibodies recognizing phospho-p44/42 MAP kinase (Thr202/Tyr204) (#9101, Cell Signaling), IκBα (#9242, Cell Signaling), or phospho-IκBα (Ser32) (#9241, Cell Signaling), actin (A-2066, Sigma), IRAK1 (F-4, Santa Cruz Biotechnology), IRAK4 (Acris), MALT1 (H-300, Santa Cruz Biotechnology) and secondary horseradish peroxidase-coupled antibodies. Monosialotetrahexosylganglioside (GM1) was visualized with the FITC-conjugated Cholera Toxin B Subunit (C-1655, Sigma). The EMSA was performed using the Odyssey Infrared EMSA Kit and AP-1 probes from LI-COR.

### Lipid raft preparations

B-cells (6 spleens per stimulation) were purified with IMag Streptavidin Particles Plus by anti-CD43 (553269, BD Biosciences) depletion and were either left untreated or stimulated with anti-IgM for the indicated times. The cells were lysed in 750 μl cold 1% Brij lysis buffer and mixed with 750 μl 85% sucrose made with MNE buffer (25 mM MES, pH 6.5; 150 mM NaCl; 5 mM EDTA). After transfer of the lysate to the centrifuge tube, 1.5 ml 35% sucrose in MNE buffer was overlaid, then 1 ml 5% sucrose in MNE buffer. After centrifugation for 16 h at 39,000 rpm in a Sorvall Ultracentrifuge OTD-C (Ultra-Rotor Sorvall AH-650), 0.4 ml fractions were collected from the top of the gradient.

## Abbreviations

LPS: lipopolysaccharide; BM: bone marrow; TLR, Toll-like receptor; AgR: antigen receptor; BAFF: B cell activation factor of the TNF family; BCR: B cell AgR; TCR: T cell AgR; PRR: pathogen recognition receptor; NF-κB: nuclear factor-κB; CARD: caspase recruitment domain; DD: death domain; BCL10: B cell CLL/lymphoma 10; MALT1: mucosa-associated lymphoid tissue lymphoma translocation gene 1; NOD: nucleotide-binding oligomerization domain containing; TACI: transmembrane activator and calcium modulator and cyclophilin ligand interactor; TNFR: tumor necrosis factor receptor; TRAF6: tumor necrosis factor receptor-associated factor 6; TAK1: transforming growth factor β-activated kinase 1; TLR: Toll-like receptor; IL: interleukin; TIR: Toll-IL-1 receptor; TIRAP: TIR domain-containing adaptor protein; TRIF: TIR domain-containing adaptor inducing interferon-β; IRAK: IL receptor-associated kinase; RIP: receptor-interacting protein; WT: wild type; (D)KO: (double) knockout; PKC: protein kinase C; RAG: recombination activating gene; TPA: 12-O-Tetradecanoyl-phorbol 13-acetate; GM1: monosialotetrahexosylganglioside.

## Competing interests

The authors declare that they have no competing interests.

## Authors' contributions

AD designed the study, performed experiments, analyzed the data and wrote the manuscript. WWS revised the manuscript and provided final approval for publication. All authors have read and approved the final manuscript.
